# A Probiotic Combination of *Lactiplantibacillus plantarum* DM083 and *Lacticaseibacillus rhamnosus* DM163 Improves Glycemic Control and Insulin Resistance in High-Fat-Diet-Induced Obese Mice

**DOI:** 10.3390/nu18132107

**Published:** 2026-06-28

**Authors:** Jeong-Hoo Lee, Je-Hyun Eom, Seung-Jo Yang, Jiyoung Hwang, Jam-Eon Park, Seung-Hwan Park, Young-Youn Kim, Hye-Sung Kim

**Affiliations:** 1 R&D Center, DOCSMEDI Co., Ltd., Goyang 10387, Republic of Korea; dlwjdgn@appleden.com (J.-H.L.); yangsj@docsmedi.com (S.-J.Y.); ghkdwldud@appleden.com (J.H.); 2 Apple Tree Institute of Biomedical Science, Apple Tree Medical Foundation, Goyang 10387, Republic of Korea; djawpgus@appleden.com; 3 Biological Resource Center, Korea Research Institute of Bioscience and Biotechnology (KRIBB), Jeongeup 56212, Republic of Korea; 4 Apple Tree Dental Hospital, Apple Tree Medical Foundation, Goyang 10387, Republic of Korea; rladuddus@appleden.com

**Keywords:** probiotics, *Lactiplantibacillus plantarum*, *Lacticaseibacillus rhamnosus*, type 2 diabetes, insulin resistance, glucose tolerance, hepatic gluconeogenesis, gut microbiota, high-fat diet, short-chain fatty acids

## Abstract

**Background/Objectives:** Type 2 diabetes mellitus (T2DM) is closely associated with obesity, insulin resistance, and gut microbiota dysbiosis. This study investigated the effects of a probiotic combination of *Lactiplantibacillus plantarum* DM083 and *Lacticaseibacillus rhamnosus* DM163 on glycemic control, insulin resistance, gut microbiota composition, and metabolic parameters in high-fat-diet (HFD)-induced obese mice. **Methods:** Male C57BL/6 mice were fed a 60% HFD for 8 weeks and subsequently administered DM083/163 (1 × 10^9^, 5 × 10^9^, or 1 × 10^10^ CFU/day) or metformin (250 mg/kg/day) for 12 weeks. Glucose metabolism, insulin resistance, hepatic gene expression, gut microbiota composition, and fecal short-chain fatty acids (SCFAs) were evaluated. **Results:** DM083/163 supplementation at 1 × 10^10^ CFU/day significantly reduced fasting blood glucose, HbA1c, oral glucose tolerance test area under the curve, and HOMA-IR compared with the HFD control group (*p* < 0.05). Hepatic expression of the gluconeogenic genes *Pck1* and *G6pc* was significantly downregulated, accompanied by reduced hepatic and serum TNF-α levels. Gut microbiota analysis revealed significant overall differences in beta diversity across groups (PERMANOVA, R^2^ = 0.262, *p* = 0.001), driven primarily by diet, with a trend toward a reduced *Bacillota*/*Bacteroidota* ratio in the high-dose group. **Conclusions:** DM083/163 supplementation improved glycemic control and insulin resistance in HFD-induced obese mice. These effects were associated with suppression of hepatic gluconeogenesis, attenuation of inflammation, and increased SCFA production, findings that are consistent with, but do not establish, modulation of the gut–liver axis. These findings support the potential use of DM083/163 as a probiotic intervention for obesity-associated T2DM.

## 1. Introduction

T2DM currently affects approximately 529 million adults worldwide and is projected to exceed 1.31 billion cases by 2050, representing one of the most pressing public health challenges of our era [1]. This trajectory is inseparably linked to rising rates of diet-induced obesity: sustained exposure to calorie-dense, high-fat diets promotes ectopic lipid deposition, systemic low-grade inflammation, and progressive impairment of hepatic glucose regulation—a triad that collectively accelerates the transition from insulin resistance to overt T2DM [2,3]. Central to this pathological cascade is the dysregulation of hepatic gluconeogenesis; chronically elevated activity of phosphoenolpyruvate carboxykinase (PEPCK) and glucose-6-phosphatase (G6Pase) sustains fasting hyperglycemia even when peripheral glucose utilization is impaired. Metformin, the first-line pharmacological agent for T2DM, principally suppresses these gluconeogenic enzymes, yet its long-term tolerability is limited by gastrointestinal side effects and contraindications in patients with renal insufficiency, underscoring the need for complementary therapeutic strategies [4,5].

A defining feature of obesity-associated T2DM is chronic low-grade inflammation, which functions as a proximal driver of insulin resistance rather than merely a secondary correlate. Adipose tissue-derived tumor necrosis factor-α (TNF-α) directly impairs insulin receptor substrate-1 (IRS-1) function by promoting inhibitory serine phosphorylation, thereby attenuating the downstream PI3K/Akt signaling cascade essential for glucose uptake [6]. In the liver, transforming growth factor-β1 (TGF-β1) activates Smad-dependent fibrogenic pathways and further disrupts hepatic insulin signaling, linking metabolic inflammation to the initiation of non-alcoholic fatty liver disease [7]. Notably, TNF-α and TGF-β1 have been shown to act in concert to sustain hepatic insulin resistance: TNF-α-driven IRS-1 serine phosphorylation impairs downstream Akt activation, while TGF-β1-mediated Smad3 signaling independently suppresses insulin receptor expression, creating overlapping mechanisms of hepatic glucose dysregulation that are not fully addressed by gluconeogenesis inhibition alone.

Gut microbial dysbiosis has emerged as an upstream amplifier of this inflammatory cascade. In obese and diabetic individuals, the gut microbiota is consistently characterized by an elevated *Bacillota*-to-*Bacteroidota* ratio, reduced phylogenetic diversity, and compromised epithelial barrier integrity—changes that collectively promote translocation of lipopolysaccharide into the systemic circulation, triggering low-grade endotoxemia and hepatic inflammatory activation [8,9]. Concomitantly, dysbiosis is associated with diminished production of short-chain fatty acids (SCFAs)—particularly acetate, propionate, and butyrate—microbial metabolites that regulate pancreatic insulin secretion, hepatic lipid metabolism, and intestinal barrier function through activation of free fatty acid receptors [10]. These observations position microbiota remodeling as a mechanistically plausible target for simultaneously addressing dysbiosis, systemic inflammation, and impaired glucose homeostasis.

Among microbiota-targeted interventions, probiotic supplementation with *Lactiplantibacillus plantarum* and *Lacticaseibacillus rhamnosus* strains has attracted particular interest given their documented capacity to attenuate intestinal inflammation, strengthen epithelial barrier integrity, and beneficially shift gut microbial composition [11,12]. Preclinical studies have demonstrated that individual strains from these species reduce fasting blood glucose and improve glucose tolerance in HFD-fed rodents; however, the antidiabetic effects of these species are typically studied in isolation, and whether combining strains with complementary functional properties provides enhanced metabolic benefits compared with individual strains has not been directly examined. Probiotic effects are markedly strain-specific, necessitating independent experimental evaluation of each novel strain combination [13,14].

In the present study, we investigated the antidiabetic potential of a novel probiotic combination comprising *Lactiplantibacillus plantarum* DM083 and *Lacticaseibacillus rhamnosus* DM163 (DM083/163). DM083 was identified through a large-scale in vivo functional screen using a high-sucrose diet-induced *Drosophila melanogaster* model, in which it demonstrated anti-hyperglycemic activity (unpublished data). DM163 was selected based on its previously identified anti-inflammatory properties during strain screening and characterization. Both strains were originally isolated from human tongue-coating samples, an ecological niche that remains relatively underexplored as a source of probiotic candidates for metabolic disorders. The combination of a glucose-lowering strain (DM083) and an anti-inflammatory strain (DM163) was intended to target two mechanistically complementary drivers of T2DM pathogenesis. In contrast to previous studies, which have largely evaluated individual gut-derived strains in isolation, the present work assesses a rationally paired, tongue-coating-derived two-strain combination across three doses against a metformin comparator, and directly verifies the gastrointestinal presence of the administered strains by strain-specific qPCR. We evaluated the effects of DM083/163 at three dose levels on glycemic control, insulin resistance, hepatic gluconeogenic and inflammatory gene expression, gut microbiota composition, and fecal SCFA production in HFD-induced obese C57BL/6 mice, with the aim of elucidating the gut–liver axis mechanisms underlying its antidiabetic activity.

## 2. Materials and Methods

### 2.1. Probiotic Strains and Preparation

*Lactiplantibacillus plantarum* DM083 and *Lacticaseibacillus rhamnosus* DM163 were originally recovered from tongue coating samples collected from a healthy Korean adult and a healthy Korean infant, respectively. Strain identity was confirmed by 16S rRNA gene sequencing in combination with whole-genome sequencing (WGS), and both isolates were subsequently lodged at the Korean Collection for Type Cultures under accession numbers KCTC14935BP (DM083) and KCTC15333BP (DM163). Safety attributes including hemolytic activity and antibiotic susceptibility profiles were assessed prior to use. Commercially manufactured lyophilized powders of DM083 and DM163 were stored at −20 °C until use. Prior to administration, viable cell counts were determined by serial dilution and plate counting on MRS agar, and target doses of 1 × 10^9^, 5 × 10^9^, and 1 × 10^10^ CFU/day were prepared accordingly. The selected dose range (1 × 10^9^, 5 × 10^9^, and 1 × 10^10^ CFU/day) was based on preliminary in-house screening data and previous studies reporting metabolic and anti-obesity effects of *Lactiplantibacillus plantarum* strains in HFD-induced obese mice, which have employed probiotic doses within a comparable range [15,16,17]. Accordingly, three dose levels were selected to evaluate potential dose-dependent efficacy within a biologically relevant range. The reported CFU values represent viable cell counts at the time of preparation. The two strains were mixed at a 1:1 CFU ratio and suspended in sterile phosphate-buffered saline (PBS; Enzynomics, Daejeon, Republic of Korea) immediately before administration. The stated doses represent the total combined dose per mouse per day, with each strain contributing 50% of the total CFU. The freshly prepared suspension was administered by oral gavage immediately after preparation, while vehicle control animals received an equal volume of PBS. Viability of the freeze-dried preparations was periodically verified throughout the study.

### 2.2. Animals and Experimental Design

Four-week-old male C57BL/6 mice of specific pathogen-free (SPF) grade were obtained from Samtako Bio Korea (Osan, Republic of Korea). The animals were maintained at 25 ± 2 °C and 50–60% relative humidity under a 12 h light/dark cycle, with food and water provided *ad libitum* throughout the study. All procedures involving animals followed institutional and national guidelines and received prior approval from the Institutional Animal Care and Use Committee (IACUC) of the KOSA BIO R&D Center, Namyangju, Republic of Korea (approval no. KSBRC-IACUC-24001, approved on 18 April 2024). Following one week of acclimation, obesity and associated metabolic dysfunction were induced by feeding a 60 kcal% high-fat diet (HFD; D12492, Research Diets, Inc., New Brunswick, NJ, USA) for eight weeks. Age-matched controls were maintained on a 10 kcal% normal diet (ND; D12450K, Research Diets, Inc.) over the same period. Upon completion of the induction phase, animals were stratified by body weight and reassigned into six groups (*n* = 10 each): ND (normal diet + PBS vehicle), HFD (high-fat diet + PBS vehicle), MET (HFD + metformin, 250 mg/kg/day), DM083/163-L (HFD + DM083/163 at 1 × 10^9^ CFU/day), DM083/163-M (HFD + DM083/163 at 5 × 10^9^ CFU/day), and DM083/163-H (HFD + DM083/163 at 1 × 10^10^ CFU/day). Daily oral gavage was performed for 12 consecutive weeks, with each animal receiving a 300 μL bolus of either the probiotic suspension or an equivalent volume of PBS. Body weight was monitored weekly during the intervention period. Feed intake was monitored daily by measuring the difference between the amount provided and the amount remaining, with feed provided in measured quantities to minimize spillage. At the end of the 12-week treatment, animals were fasted for 12 h and deeply anesthetized with isoflurane (3–5%). Whole blood was collected by cardiac puncture and allowed to stand on ice for at least 30 min, followed by centrifugation (3000 rpm, 10 min, 4 °C) to obtain serum. The serum was immediately aliquoted and stored at −80 °C until analysis. Liver, kidney, spleen, and adipose tissue depots (subcutaneous, visceral, and perirenal fat) were excised, rinsed with ice-cold 0.9% saline, weighed, and stored at −80 °C until further processing. Animals were subsequently euthanized by cervical dislocation.

### 2.3. Oral Glucose Tolerance Test (OGTT)

Glucose tolerance was evaluated at the conclusion of the 12-week intervention period. Following an overnight fast of 12 h, a glucose bolus (2 g/kg body weight) was delivered by oral gavage to each animal. Capillary blood was collected from the tail vein at baseline (0 min) and at 30, 60, 90, and 120 min after gavage, and glucose concentrations were determined in duplicate using an Accu-Chek Instant glucometer (Roche Diabetes Care GmbH, Mannheim, Germany). The total glycemic excursion over the 120 min sampling window was estimated as the area under the curve (AUC) using the trapezoidal rule.

### 2.4. Biochemical Analysis

Fasting glucose was quantified with the Accu-Chek Instant glucometer (Roche Diabetes Care GmbH). Circulating insulin was determined in serum samples using a Mouse Ultrasensitive Insulin ELISA kit (ALPCO, Salem, NH, USA), and HbA1c was assayed with the Mouse Hemoglobin A1c ELISA kit (ab285317; Abcam, Cambridge, UK), each according to the manufacturer’s recommended protocol. The degree of insulin resistance was estimated through the homeostasis model assessment (HOMA-IR) [18], computed as:

HOMA-IR = (fasting glucose [mg/dL] × fasting insulin [μU/mL])/405

### 2.5. Serum Cytokine Analysis

Serum TNF-α concentrations were quantified using a multiplex electrochemiluminescence immunoassay system (V-PLEX Proinflammatory Panel 1, Meso Scale Diagnostics, Rockville, MD, USA) according to the manufacturer’s instructions.

### 2.6. Gut Microbiota Analysis

Sequencing library preparation and bioinformatic processing were carried out essentially as previously described, with minor modifications. Briefly, total microbial DNA was recovered from fecal pellets through mechanical lysis using a bead-beating procedure. The hypervariable V3–V4 region of the bacterial 16S rRNA gene was PCR-amplified, and the resulting amplicons were converted into sequencing libraries. Library concentration was determined fluorometrically with the Quant-iT PicoGreen dsDNA Assay Kit (Invitrogen, Waltham, MA, USA), while size distribution and integrity were verified on an Agilent 2100 Bioanalyzer (Agilent Technologies, Santa Clara, CA, USA). Residual short fragments were eliminated by magnetic bead-based purification using the CleanPCR system (CleanNA, Alphen aan den Rijn, The Netherlands). Pooled libraries were sequenced as 2 × 300 bp paired-end reads on an Illumina NextSeq 1000 instrument with the NextSeq P2 reagent kit (600 cycles; Illumina, San Diego, CA, USA). Demultiplexed paired-end reads were imported into R (v4.3.0) and processed with the DADA2 pipeline (v1.26) [19]. Read trimming was set to 240 bp for the forward and 160 bp for the reverse reads, with maxEE thresholds of 2 and 5, truncQ = 2, and concurrent removal of PhiX-derived sequences. Denoising was performed in pseudo-pool mode (pool = “pseudo”) prior to read merging and construction of the amplicon sequence variant (ASV) table. ASVs below 300 bp in length were filtered out, and putative chimeras were eliminated by the consensus algorithm of remove BimeraDenovo. Libraries yielding fewer than 10,000 high-quality reads were not retained for downstream comparisons. Taxonomy was assigned against the SILVA 138.1 reference (silva_nr99_v138.1_train_set) via the assignTaxonomy function with min Boot = 80, followed by species-level annotation using addSpecies with the silva_species_assignment_v138.1 dataset. Community-level analyses were conducted in R (v4.3.0) using the phyloseq and vegan packages. Within-sample richness and evenness were summarized through the Shannon index, observed ASV counts, and the Chao1 estimator. Between-sample dissimilarities were computed on Bray–Curtis distances, and the statistical significance of group-wise differences in community structure was tested by permutational multivariate analysis of variance (PERMANOVA) with 999 permutations. All raw sequence reads generated in the present study are accessible from the NCBI Sequence Read Archive (SRA) under BioProject ID PRJNA1453943.

### 2.7. Short-Chain Fatty Acid (SCFA) Analysis

Fecal SCFA profiles were obtained by gas chromatography. Aliquots of feces were weighed into tubes containing glass beads, mixed with 0.5 mL of acetyl chloride/methanol (5:100, *v*/*v*; acetyl chloride, Sigma-Aldrich, St. Louis, MO, USA; methanol, Merck, Darmstadt, Germany), and homogenized in a bead beater, after which the mixtures were heated at 80 °C for 1 h. Liquid–liquid extraction was then performed with 0.5 mL of n-hexane (Burdick & Jackson, Muskegon, MI, USA), and the resulting upper organic layer was filtered prior to chromatographic injection. Quantification of acetic, propionic, butyric, and valeric acids was carried out on a Shimadzu GC-2010 Plus instrument (Kyoto, Japan) equipped with a DB-23 capillary column (60 m × 0.25 mm; Agilent Technologies, Santa Clara, CA, USA) and flame ionization detection at 260 °C. Injection volume was 1 μL with a 10:1 split ratio at 250 °C, using nitrogen as the carrier gas. The oven temperature program proceeded as follows: 60 °C held for 5 min; ramping to 100 °C at 20 °C/min and held for 2 min; further ramping to 200 °C at 25 °C/min and held for 5 min. External calibration curves were prepared using authentic standards of acetic acid (Samchun, Pyeongtaek, Republic of Korea), propionic acid, butyric acid, and valeric acid (Sigma-Aldrich, St. Louis, MO, USA) for quantitation. SCFA concentrations were normalized to the wet weight of fecal samples and expressed as μg/g wet feces. Quantification was performed using external calibration curves generated from authentic standards of acetic, propionic, butyric, and valeric acids over a concentration range of 2.441–156.25 μg/mL using a seven-point, two-fold serial dilution scheme. Validation parameters, including calibration range, coefficients of determination (R^2^), limits of detection (LOD), and limits of quantification (LOQ), are summarized in Table S1. Two independent calibration runs demonstrated good analytical reproducibility, with inter-day slope relative standard deviations ranging from 1.26% to 7.84%. Although no internal standard was employed, the calibration performance and reproducibility of the analytical method, together with the fact that the majority of measured fecal SCFA concentrations fell within the validated quantification range, support the reliability of SCFA quantification.

### 2.8. Hepatic Gene Expression Analysis

Hepatic transcript abundance was assessed essentially as previously reported, with adaptations for the mouse-specific targets of this study. Total RNA from liver tissue was isolated with the Hybrid-R™ kit (GeneAll Biotechnology, Seoul, Republic of Korea), and RNA quality was verified by spectrophotometric assessment of A260/A280 ratios prior to reverse transcription. First-strand cDNA was generated using the PrimeScript™ RT Reagent Kit (Takara Bio Inc., Shiga, Japan). Quantitative PCR was carried out on a QuantStudio™ 3 instrument (Applied Biosystems, Foster City, CA, USA) with TOPreal™ qPCR 2× PreMIX SYBR Green chemistry (Enzynomics, Daejeon, Republic of Korea). Targets included *Pck1* (PEPCK), *G6pc* (G6Pase), *Tnf* (TNF-α), and *Tgfb1* (TGF-β1). Cycling conditions consisted of an initial denaturation step at 95 °C for 10 min, followed by 40 amplification cycles (95 °C, 10 s; 60 °C, 15 s; 72 °C, 20 s). Relative expression values were derived using the 2^−ΔΔCt^ approach with *Gapdh* serving as the endogenous reference [20]. The primer sequences and amplicon sizes used for RT-qPCR are listed in Table 1.

### 2.9. Strain-Targeted qPCR Analysis

To assess the enrichment of DM083- and DM163-associated signals in fecal samples, qPCR was performed using primers designed from whole-genome sequence information of *Lactiplantibacillus plantarum* DM083 and *Lacticaseibacillus rhamnosus* DM163. Total DNA extracted for gut microbiota analysis was used as template DNA for qPCR analysis. Amplification was performed using a QuantStudio™ 3 instrument (Applied Biosystems, Foster City, CA, USA) with TOPreal™ qPCR 2× PreMIX SYBR Green chemistry (Enzynomics, Daejeon, Republic of Korea). Cycling conditions consisted of an initial denaturation at 95 °C for 10 min, followed by 40 amplification cycles (95 °C, 10 s; 64 °C, 20 s; 72 °C, 20 s). Ct values were used to compare target-associated signal abundance among groups. Primer sequences and amplicon information are provided in Table S2.

### 2.10. Statistical Analysis

**Figure 1:**
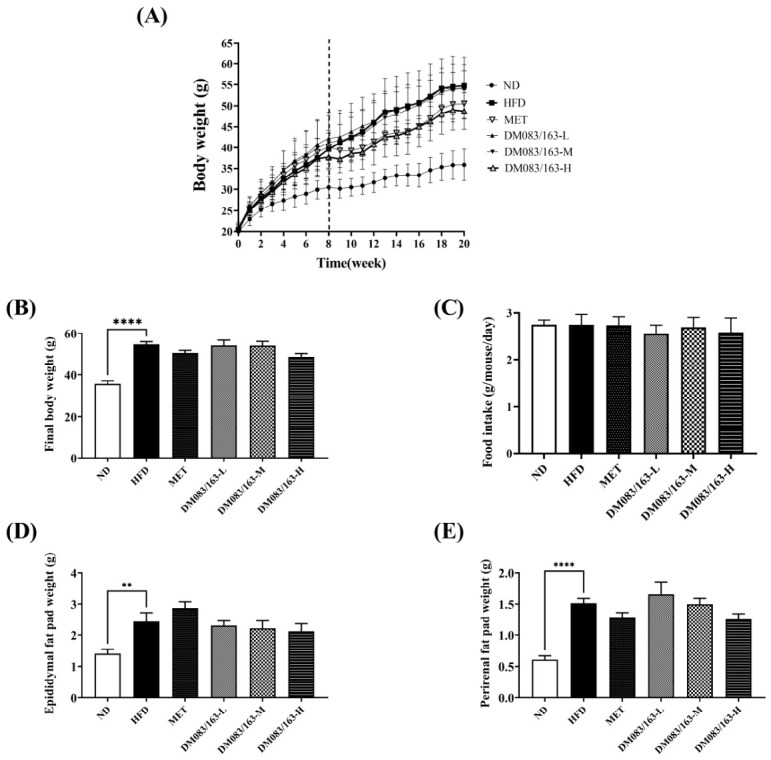
Effects of DM083/163 probiotic supplementation on body weight and food intake in high-fat diet-fed C57BL/6 mice. (**A**) Weekly body weight changes over 20 weeks. The dashed vertical line indicates the start of probiotic administration (week 8). (**B**) Final body weight at week 20. (**C**) Average daily food intake during the experimental period. Data are expressed as mean ± SD (*n* = 8 per group). Statistical significance was determined by two-way repeated measures ANOVA with Dunnett’s post hoc test for (**A**), and one-way ANOVA with Dunnett’s post hoc test for (**B**) and (**C)**. In (**B**), **** *p* < 0.0001 (ND versus HFD) and ns denotes no significant difference between the HFD and DM083/163-H groups. ND, normal diet; HFD, high-fat diet; MET, metformin (250 mg/kg/day); DM083/163-L, 1 × 10^9^ CFU/day; DM083/163-M, 5 × 10^9^ CFU/day; DM083/163-H, 1 × 10^10^ CFU/day. DM083/163 consists of *Lactiplantibacillus plantarum* DM083 and *Lacticaseibacillus rhamnosus* DM163. (**D**) Epididymal fat pad weight. (**E**) Perirenal fat pad weight. Data are expressed as mean ± SD (*n* = 8 per group). Statistical significance was determined by one-way ANOVA with Dunnett’s post hoc test. ** *p* < 0.01, **** *p* < 0.0001 versus ND group.

**Figure 2:**
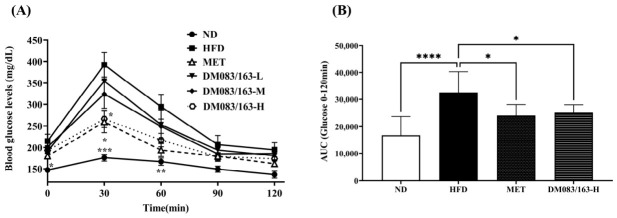
Effects of DM083/163 probiotic supplementation on oral glucose tolerance in high-fat diet-induced obese mice. (**A**) Blood glucose levels during the oral glucose tolerance test (OGTT) performed at week 20. Glucose (2 g/kg body weight) was administered by oral gavage following a 12 h fast, and blood glucose was measured from the tail vein at 0, 30, 60, 90, and 120 min. (**B**) Area under the curve (AUC) of blood glucose levels during the week 20 OGTT (0 – 120 min), calculated using the trapezoidal method. Only the ND, HFD, MET, and DM083/163-H groups are shown in panel (**B**); data for the lower-dose groups (DM083/163-L and DM083/163-M) are presented in panel (**A**). Data are presented as mean ± SD (*n* = 8 per group). Statistical significance was determined by two-way repeated-measures ANOVA followed by Dunnett’s post hoc test for panel (**A**) and one-way ANOVA followed by Dunnett’s post hoc test for panel (**B**). * *p* < 0.05, ** *p* < 0.01, *** *p* < 0.001, **** *p* < 0.0001 versus HFD group. ND, normal diet; HFD, high-fat diet; MET, metformin (250 mg/kg/day); DM083/163-L, 1 × 10^9^ CFU/day; DM083/163-M, 5 × 10^9^ CFU/day; DM083/163-H, 1 × 10^10^ CFU/day. DM083/163 consists of *Lactiplantibacillus plantarum* DM083 and *Lacticaseibacillus rhamnosus* DM163.

The sample size of *n* = 10 animals per group was determined on the basis of previous HFD-induced T2DM mouse studies, in which group sizes of 8–10 animals were reported to provide sufficient sensitivity to detect biologically meaningful differences in key metabolic parameters. Although a formal a priori power calculation was not performed, this group size is consistent with the established standard for diet-induced obesity models, and significant differences were detected for the principal glycemic outcomes under the present design. During the study, a small number of animals were lost owing to mortality, and certain assays could not be performed on all animals owing to insufficient blood or tissue samples. Accordingly, the final number of biological replicates analyzed was eight per group for body weight, glycemic, and biochemical parameters, and ten per group for gut microbiota (16S rRNA amplicon sequencing), fecal SCFA, and strain-specific qPCR analyses; the exact replicate number for each measurement is indicated in the corresponding figure legend. All statistical computations were performed in GraphPad Prism v10.0 (GraphPad Software, San Diego, CA, USA), with microbiome-related analyses (alpha diversity metrics, Bray–Curtis distances, and PERMANOVA) executed in R v4.3.0 (R Foundation for Statistical Computing, Vienna, Austria) under the phyloseq and vegan frameworks. Quantitative results are presented as mean ± standard deviation (SD). Data were assessed for normality and homogeneity of variance prior to parametric testing. Fecal SCFA values were log-transformed before parametric testing to better approximate normality. Repeated measurements collected longitudinally on the same animals—namely weekly body weights from week 0 through week 20 (Figure 1A) and OGTT glucose curves obtained at week 12 (Figure 2A)—were evaluated by two-way repeated-measures ANOVA with Dunnett’s post hoc comparisons referenced to the HFD group. For cross-sectional endpoints, including final body weight, daily food intake, OGTT-derived AUC, fasting glucose, HbA1c, serum insulin, HOMA-IR, hepatic *Pck1*/*G6pc*/*Tnf*/*Tgfb1* expression, serum TNF-α, and log-transformed fecal SCFAs, group-wise comparisons were carried out by one-way ANOVA with Dunnett’s post hoc test against the HFD reference. Alpha diversity indices were compared by Kruskal–Wallis testing followed by Dunn’s pairwise procedure with Benjamini–Hochberg correction. Differences in microbial community structure (beta diversity) were tested by PERMANOVA on Bray–Curtis distances using 999 permutations. Two-tailed *p*-values below 0.05 were considered statistically significant, and significance levels were denoted as * *p* < 0.05, ** *p* < 0.01, *** *p* < 0.001, **** *p* < 0.0001 versus HFD unless noted otherwise.

## 3. Results

### 3.1. Effects of DM083/163 Supplementation on Body Weight and Food Intake

HFD feeding induced a significant and progressive increase in body weight compared to the ND group throughout the experimental period (Figure 1A). At week 20, final body weight in the DM083/163 1 × 10^10^ CFU/day group tended to be lower than that of the HFD control group, although the difference did not reach statistical significance (Figure 1B); no significant differences were observed at the lower doses (1 × 10^9^ and 5 × 10^9^ CFU/day). The metformin-treated group did not show a statistically significant reduction in final body weight. Daily food intake did not differ significantly among the experimental groups throughout the treatment period (Figure 1C). Food intake was recorded at the cage level and expressed as the average daily intake per animal without correction for feed spillage. In addition, although HFD feeding significantly increased epididymal (*p* < 0.01) and perirenal (*p* < 0.0001) adipose tissue weights compared with the ND group (Figure 1D,E), no significant differences in either fat depot were observed among the HFD-fed groups, including the DM083/163-H group.

### 3.2. DM083/163 Improved Glucose Tolerance, Fasting Blood Glucose, and HbA1c

To evaluate the effects of DM083/163 on glucose homeostasis and insulin sensitivity, oral glucose tolerance, fasting glycemic parameters, serum insulin levels, and HOMA-IR were assessed following 12 weeks of probiotic supplementation.

#### 3.2.1. Oral Glucose Tolerance Test

To evaluate the effect of DM083/163 supplementation on postprandial glucose metabolism, an OGTT was performed after 12 weeks of treatment. The HFD control group exhibited markedly elevated blood glucose levels throughout the test compared to the ND group, with peak levels at 30 min (*p* < 0.001; Figure 2A). Among the treatment groups, the metformin-treated group showed significant reductions in blood glucose at both 30 min (*p* < 0.05) and 60 min (*p* < 0.05) compared to the HFD control group. DM083/163 supplementation at 1 × 10^10^ CFU/day significantly attenuated the glucose excursion at 30 min (*p* < 0.05) compared to the HFD control group, with a trend toward reduction at subsequent time points that did not reach statistical significance. The glucose AUC from 0 to 120 min was significantly reduced in both the metformin group (*p* < 0.01) and the 1 × 10^10^ CFU/day group (*p* < 0.01) compared to the HFD control, with no significant difference observed between these two groups (Figure 2B).

#### 3.2.2. DM083/163 Improved Fasting Glycemic Parameters and Insulin Resistance

**Figure 3:**
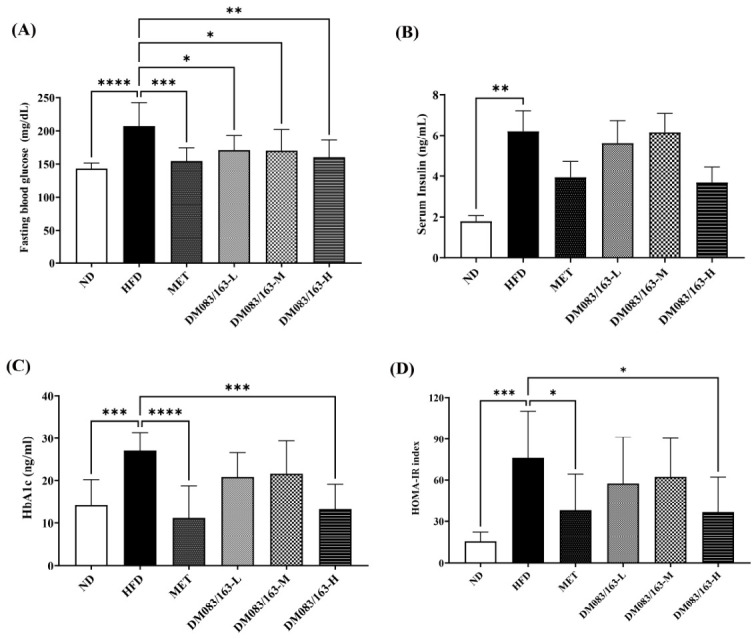
Effects of DM083/163 probiotic supplementation on fasting blood glucose, serum insulin, HbA1c, and insulin resistance in high-fat diet-induced obese mice. (**A**) Fasting blood glucose levels at the end of the experimental period. (**B**) Serum insulin concentrations. (**C**) Glycated hemoglobin (HbA1c) levels. (**D**) Homeostasis model assessment of insulin resistance (HOMA-IR), calculated as [fasting glucose (mg/dL) × fasting insulin (μU/mL)]/405. Data are expressed as mean ± SD (*n* = 8 per group). Statistical significance was determined by one-way ANOVA with Dunnett’s post hoc test. * *p* < 0.05, ** *p* < 0.01, *** *p* < 0.001, **** *p* < 0.0001 versus HFD group. ND, normal diet; HFD, high-fat diet; MET, metformin (250 mg/kg/day); DM083/163-L, 1 × 10^9^ CFU/day; DM083/163-M, 5 × 10^9^ CFU/day; DM083/163-H, 1 × 10^10^ CFU/day. DM083/163 consists of *Lactiplantibacillus plantarum* DM083 and *Lacticaseibacillus rhamnosus* DM163.

Fasting blood glucose levels were significantly elevated in the HFD control group compared with the ND group (*p* < 0.0001; Figure 3A). DM083/163 supplementation significantly reduced fasting blood glucose at 1 × 10^9^ (*p* < 0.05), 5 × 10^9^ (*p* < 0.05), and 1 × 10^10^ CFU/day (*p* < 0.01) compared with the HFD control group, while the metformin-treated group also showed a significant reduction (*p* < 0.001). Serum insulin levels were significantly elevated in the HFD control group compared with the ND group (*p* < 0.01; Figure 3B), indicating the development of hyperinsulinemia. Although the MET and DM083/163-H groups exhibited lower mean serum insulin concentrations than the HFD control group, these differences did not reach statistical significance (*p* = 0.225 and *p* = 0.151, respectively). HbA1c levels were significantly higher in the HFD control group than in the ND group (*p* < 0.001; Figure 3C). Both the metformin-treated group (*p* < 0.0001) and the DM083/163-H group (*p* < 0.001) significantly reduced HbA1c compared with the HFD control group, with the high-dose group achieving levels comparable to those of the ND group. Consistent with the changes in fasting glucose, HOMA-IR values were significantly elevated in the HFD control group compared with the ND group (*p* < 0.001; Figure 3D). DM083/163 supplementation reduced HOMA-IR, with a significant effect observed in the DM083/163-H group (*p* < 0.05 versus HFD). The metformin-treated group also showed a significant reduction in HOMA-IR compared with the HFD group (*p* < 0.05). These findings indicate that the improvement in HOMA-IR was driven primarily by the reduction in fasting blood glucose rather than by a significant change in circulating insulin.

### 3.3. DM083/163 Suppressed Hepatic Gluconeogenesis and Inflammation

To investigate potential mechanisms underlying the improvements in glycemic control and insulin resistance, hepatic expression of key genes involved in gluconeogenesis and inflammation was evaluated. Because excessive hepatic glucose production and chronic low-grade inflammation are central contributors to obesity-associated T2DM, we examined the effects of DM083/163 on these pathways.

#### 3.3.1. Hepatic Gluconeogenic Gene Expression

**Figure 4:**
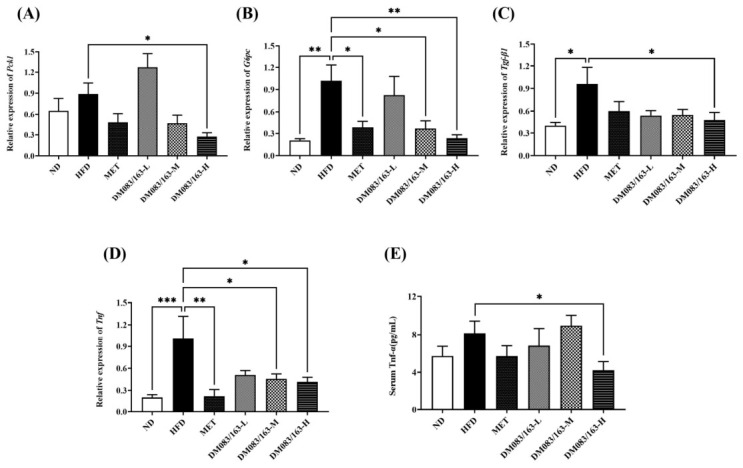
Effects of DM083/163 probiotic supplementation on hepatic gluconeogenic and inflammatory markers in high-fat diet-induced obese mice. (**A**) Relative expression of *Pck1* (PEPCK). (**B**) Relative expression of *G6pc* (G6Pase). (**C**) Relative expression of *Tgfb1* (TGF-β1). (**D**) Relative expression of *Tnf* (TNF-α). (**E**) Serum tumor necrosis factor-α (TNF-α) concentration. Gene expression levels in panels (**A**–**D**) were normalized to GAPDH using the 2^−ΔΔCt^ method and expressed relative to the HFD group. Data are expressed as mean ± SD (*n* = 8 per group). Statistical significance was determined by one-way ANOVA with Dunnett’s post hoc test. * *p* < 0.05, ** *p* < 0.01, *** *p* < 0.001 versus HFD group. ND, normal diet; HFD, high-fat diet; MET, metformin (250 mg/kg/day); DM083/163-L, 1 × 10^9^ CFU/day; DM083/163-M, 5 × 10^9^ CFU/day; DM083/163-H, 1 × 10^10^ CFU/day. DM083/163 consists of *Lactiplantibacillus plantarum* DM083 and *Lacticaseibacillus rhamnosus* DM163. HbA1c, glycated hemoglobin; HOMA-IR, homeostasis model assessment of insulin resistance.

Hepatic *Pck1* (PEPCK) expression showed an increasing trend in the HFD control group compared to the ND group, although this difference did not reach statistical significance (Figure 4A). DM083/163-H significantly reduced *Pck1* expression compared with the HFD control group (*p* < 0.05). Hepatic *G6pc* (G6Pase) expression was significantly elevated in the HFD control group compared with the ND group (*p* < 0.01; Figure 4B) and was significantly reduced by MET (*p* < 0.05), DM083/163-M (5 × 10^9^ CFU/day; *p* < 0.05), and DM083/163-H (*p* < 0.01). These findings suggest that DM083/163 suppresses hepatic gluconeogenic gene expression, particularly at higher doses.

#### 3.3.2. Hepatic and Systemic Inflammatory Markers

Hepatic *Tgfb1* (TGF-β1) expression was significantly elevated in the HFD control group relative to the ND group (*p* < 0.05; Figure 4C) and was significantly reduced by DM083/163 at 1 × 10^10^ CFU/day (*p* < 0.05). Hepatic *Tnf* (TNF-α) expression in liver tissue was significantly elevated in the HFD control group relative to the ND group (*p* < 0.001; Figure 4D). DM083/163 at 5 × 10^9^ and 1 × 10^10^ CFU/day significantly reduced hepatic *Tnf* expression compared with the HFD control group (*p* < 0.05 for both), while MET also showed a significant reduction (*p* < 0.01). Serum TNF-α concentrations were also significantly reduced in the DM083/163-H group compared with the HFD control group (*p* < 0.05; Figure 4E), supporting the anti-inflammatory effects of DM083/163.

### 3.4. Effects of DM083/163 on Gut Microbiota Composition

#### 3.4.1. Community Diversity Analysis

**Figure 5:**
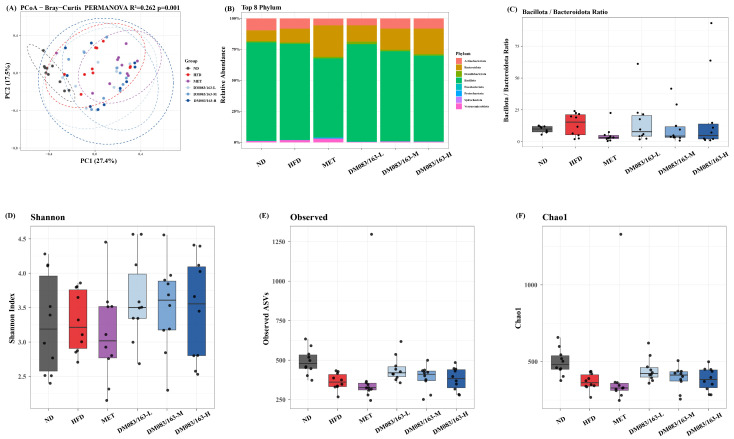
Effects of DM083/163 probiotic supplementation on gut microbiota composition in high-fat diet-induced obese mice. (**A**) Principal coordinates analysis (PCoA) plot based on Bray–Curtis dissimilarity. Each point represents an individual sample. Dashed ellipses indicate 95% confidence intervals for each group. PERMANOVA R^2^ = 0.262, *p* = 0.001. PC1 and PC2 represent the first and second principal coordinates, with the percentage of total variance explained shown in parentheses. (**B**) Relative abundance of the top 8 bacterial phyla across experimental groups. (**C**) *Bacillota*/*Bacteroidota* ratio shown as box plots. (**D**) Shannon diversity index. (**E**) Observed ASVs. (**F**) Chao1 richness estimator. Microbiota analyses were performed on *n* = 10 per group; each dot represents an individual biological replicate. For box plots (**C**–**F**), the center line indicates the median; box boundaries indicate the interquartile range (IQR); whiskers extend to 1.5 × IQR. ND, normal diet; HFD, high-fat diet; MET, metformin (250 mg/kg/day); DM083/163-L, 1 × 10^9^ CFU/day; DM083/163-M, 5 × 10^9^ CFU/day; DM083/163-H, 1 × 10^10^ CFU/day. DM083/163 consists of *Lactiplantibacillus plantarum* DM083 and *Lacticaseibacillus rhamnosus* DM163.

PCoA based on Bray–Curtis dissimilarity demonstrated clear separation of microbial communities among experimental groups (Figure 5A). PERMANOVA indicated significant differences in community structure across all six groups (R^2^ = 0.262, *p* = 0.001). However, pairwise comparisons showed that this overall difference was driven primarily by the dietary contrast, as all ND-versus-HFD-based comparisons were highly significant (R^2^ = 0.281–0.404, *p* = 0.001), whereas the high-dose probiotic group did not differ significantly from the HFD control group (HFD vs. DM083/163-H, R^2^ = 0.081, *p* = 0.102). Among the treatment groups, only metformin produced a community structure significantly distinct from the HFD control (R^2^ = 0.140, *p* = 0.006). These results indicate that HFD feeding was the dominant determinant of gut microbial community structure, and that DM083/163 supplementation did not significantly reshape overall beta diversity relative to the HFD control. Alpha diversity indices, including the Shannon diversity index, observed ASVs, and Chao1 richness estimator, showed a trend toward reduced microbial diversity and richness in HFD-fed mice compared with the ND group. Partial recovery was observed in the probiotic-treated groups, particularly in the DM083/163-H group; however, none of these differences reached statistical significance (Figure 5D–F).

#### 3.4.2. Phylum-Level Composition and *Bacillota*/*Bacteroidota* Ratio

At the phylum level, HFD feeding was associated with increased relative abundance of *Bacillota* (formerly Firmicutes) and reduced *Bacteroidota* (formerly Bacteroidetes) compared to the ND group (Figure 5B). The *Bacillota*/*Bacteroidota* ratio tended to be lower in the DM083/163-H group than in the HFD control group, suggesting partial restoration of microbial balance (Figure 5C). At the genus level, distinct compositional differences were observed among the experimental groups, with changes in the relative abundance of several dominant genera, including *Allobaculum*, *Lactobacillus*, and *Limosilactobacillus* (Figure S1). It is noteworthy that the administered probiotic strains—*Lactiplantibacillus plantarum* DM083 and *Lacticaseibacillus rhamnosus* DM163—were not unambiguously assigned to their respective genera by 16S rRNA V3–V4 amplicon sequencing in the present analysis. This likely reflects the limited taxonomic resolution of the V3–V4 region for closely related members of the family Lactobacillaceae, combined with the conservative bootstrap confidence threshold (minBoot = 80) applied during taxonomic assignment, rather than the absence of these strains in the gut. The gastrointestinal presence of the administered strains was subsequently confirmed by strain-specific qPCR (Section 3.6).

### 3.5. DM083/163 Increased Fecal Short-Chain Fatty Acid Concentrations

**Figure 6:**
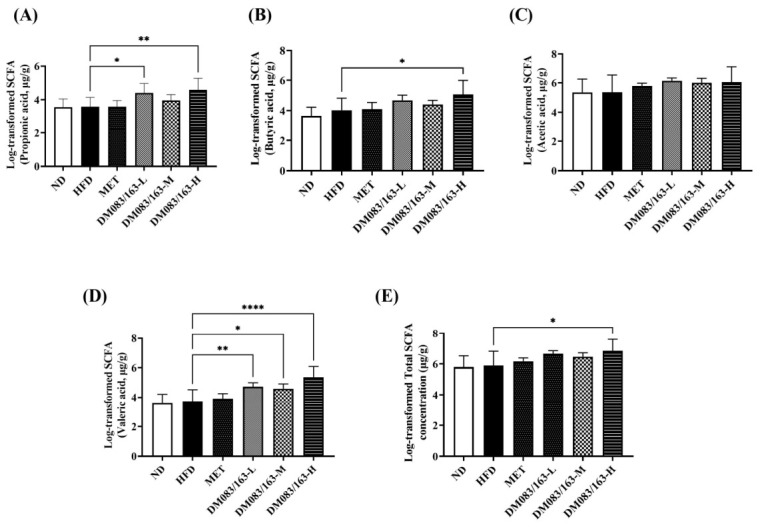
Effects of DM083/163 probiotic supplementation on fecal short-chain fatty acid(SCFA) concentrations in high-fat diet-fed C57BL/6 mice. (**A**) Propionic acid, (**B**) butyric acid, (**C**) acetic acid, (**D**) valeric acid, and (**E**) total SCFA concentrations in fecal samples. SCFA concentrations are expressed as μg/g wet feces; values were log-transformed for statistical analysis. Data are expressed as mean ± SD (*n* = 10 per group). Statistical significance was determined by one-way ANOVA with Dunnett’s post hoc test. * *p* < 0.05, ** *p* < 0.01, **** *p* < 0.0001 versus HFD group. ND, normal diet; HFD, high-fat diet; MET, metformin (250 mg/kg/day); DM083/163-L, 1 × 10^9^ CFU/day; DM083/163-M, 5 × 10^9^ CFU/day; DM083/163-H, 1 × 10^10^ CFU/day. DM083/163 consists of *Lactiplantibacillus plantarum* DM083 and *Lacticaseibacillus rhamnosus* DM163.

DM083/163 supplementation significantly increased propionic acid concentrations at 1 × 10^9^ CFU/day (*p* < 0.05) and 1 × 10^10^ CFU/day (*p* < 0.01) compared to the HFD control group (Figure 6A). Butyric acid concentrations were significantly elevated in the 1 × 10^10^ CFU/day group compared to the HFD control (*p* < 0.05; Figure 6B), although inter-individual variability was observed. In contrast, acetic acid showed no significant differences among groups (Figure 6C). Valeric acid concentrations were significantly increased in the 1 × 10^9^ (*p* < 0.01), 5 × 10^9^ (*p* < 0.05), and 1 × 10^10^ CFU/day (*p* < 0.0001) groups compared to the HFD control, suggesting a dose-dependent tendency (Figure 6D). Consistent with these individual SCFA changes, total fecal SCFA concentration was significantly elevated in the 1 × 10^10^ CFU/day group compared to the HFD control (*p* < 0.05; Figure 6E), indicating that high-dose DM083/163 supplementation enhanced overall SCFA production. These findings suggest that DM083/163-induced changes in the gut microbiota are functionally associated with increased fermentative activity and SCFA production. As the SCFA analysis was performed without an internal standard (Section 2.7), the absolute concentrations should be interpreted with this analytical limitation in mind, although method validation supported the reliability of the relative group comparisons.

### 3.6. Strain-Specific qPCR Confirmed Gastrointestinal Presence of DM083 and DM163

**Figure 7:**
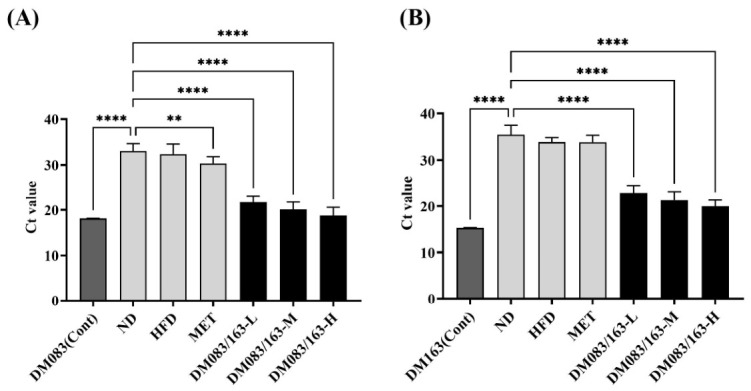
Strain-specific qPCR detection of DM083 and DM163 in fecal samples collected at study termination. (**A**) DM083-specific qPCR. (**B**) DM163-specific qPCR. Lower cycle threshold (Ct) values indicate higher abundance of strain-specific DNA. Data are presented as mean ± SD (*n* = 10 per group). Statistical significance was determined by one-way ANOVA followed by Dunnett’s multiple comparisons test using the ND group as the reference. ** *p* < 0.01, **** *p* < 0.0001 versus ND. DM083(Cont) and DM163(Cont) represent genomic DNA isolated from the corresponding probiotic strains and were included as positive controls. ND, normal diet; HFD, high-fat diet; MET, metformin (250 mg/kg/day); DM083/163-L, 1 × 10^9^ CFU/day; DM083/163-M, 5 × 10^9^ CFU/day; DM083/163-H, 1 × 10^10^ CFU/day. DM083/163 consists of *Lactiplantibacillus plantarum* DM083 and *Lacticaseibacillus rhamnosus* DM163.

To further assess the presence of the administered strains, endpoint fecal qPCR was performed using strain-specific primers designed from whole-genome sequence information. Probiotic-treated groups exhibited markedly lower Ct values than non-administered groups (ND, HFD, and MET) in both DM083- and DM163-specific assays, indicating the presence of strain-associated DNA in fecal samples following supplementation (Figure 7). These findings provide evidence of gastrointestinal exposure to the administered strains during the supplementation period, but do not by themselves establish stable colonization or persistence. Nevertheless, qPCR detection alone cannot distinguish between transient gastrointestinal passage and stable gut colonization; therefore, definitive confirmation of long-term engraftment would require longitudinal monitoring and/or shotgun metagenomic sequencing.

## 4. Discussion

The present study was designed to investigate whether the probiotic combination DM083/163 could ameliorate HFD-induced metabolic dysfunction through coordinated modulation of glucose metabolism, hepatic inflammatory responses, and the gut microbiota. The major finding was that high-dose DM083/163 improved glycemic control and insulin resistance, accompanied by suppression of hepatic gluconeogenic and inflammatory markers, alterations in gut microbial composition, and increased fecal SCFA production. These findings suggest that the antidiabetic effects of DM083/DM163 are unlikely to be explained by a single pathway, but rather by integrated regulation of the gut–liver axis.

An additional strength of the present study lies in the rationale underlying strain selection. DM083 and DM163 were originally isolated from human tongue-coating samples, an ecological niche that remains relatively underexplored as a source of probiotic candidates for metabolic disorders. DM083 was identified through a prior in vivo functional screening strategy using a high-sucrose-diet-induced Drosophila model, whereas DM163 was selected based on its previously characterized anti-inflammatory properties. Accordingly, DM083 and DM163 were selected for further evaluation as a probiotic combination in an HFD-induced obesity model. Importantly, rather than inferring strain activity solely from prior reports, we directly confirmed the gastrointestinal presence of both administered strains using strain-specific qPCR (Figure 7). This provides study-generated evidence of strain delivery to the gut, although it does not by itself establish a causal link between the strains and the observed metabolic effects.

The establishment of the HFD-induced obese mouse model was confirmed by significant increases in body weight, fasting blood glucose, HbA1c, serum insulin, and HOMA-IR in the HFD control group compared to ND mice. These findings are consistent with previous reports demonstrating that prolonged HFD feeding in C57BL/6 mice reliably induces obesity, hyperglycemia, and insulin resistance, providing a well-validated preclinical model for T2DM research [21,22]. The observation that DM083/163 at 1 × 10^10^ CFU/day showed a trend toward reduced final body weight without affecting food intake suggests a possible weight-modulating effect that, if confirmed, would be unlikely to result from appetite suppression and might instead reflect metabolic changes such as altered energy expenditure or lipid metabolism. Interestingly, the reduction in body weight was not accompanied by significant changes in epididymal or perirenal adipose tissue weights (Figure 1D,E). Furthermore, food intake was measured at the cage level rather than the individual animal level, and parameters such as food efficiency ratio, body composition, and energy expenditure were not assessed. Therefore, the mechanisms underlying the apparent body weight-modulating trend observed with DM083/163, which did not reach statistical significance, remain to be clarified. This finding is consistent with reports demonstrating that certain *Lactiplantibacillus plantarum* strains reduce adiposity through modulation of lipid absorption and adipogenesis-related gene expression [15,16].

The significant improvement in glucose tolerance observed in the DM083/163 high-dose group, as evidenced by reduced OGTT AUC and lower fasting blood glucose and HbA1c levels, represents the primary efficacy endpoint of this study. These improvements were broadly comparable to those observed in the metformin-treated group under the present experimental conditions. Similar glycemic improvements have been reported with *L. plantarum*-containing probiotics in HFD-fed rodent models, attributed to enhanced insulin signaling, reduced hepatic glucose output, and improved pancreatic β-cell function [17,23]. Furthermore, the significant reduction in HbA1c, a marker of long-term glycemic control, suggests that the beneficial effects of DM083/163 were sustained throughout the treatment period rather than representing acute glucose-lowering effects. The most pronounced effects were observed at 1 × 10^10^ CFU/day, which was the highest dose evaluated in the present study. Whether the observed benefits reach a plateau at this dose or could be further enhanced at higher doses remains to be determined and warrants investigation in future dose-escalation studies.

A key mechanistic finding of this study was the significant downregulation of hepatic PEPCK and G6Pase mRNA expression in the DM083/163 high-dose group. PEPCK and G6Pase are rate-limiting enzymes in hepatic gluconeogenesis, and their upregulation in insulin-resistant states represents a major driver of fasting hyperglycemia [24]. Metformin is known to exert its primary glucose-lowering effect through inhibition of hepatic gluconeogenesis via activation of AMP-activated protein kinase (AMPK) and suppression of the gluconeogenic gene program [5]. The observation that DM083/163 similarly suppressed PEPCK and G6Pase expression suggests a potentially overlapping mechanism of action, possibly mediated through gut-derived signals that activate hepatic AMPK or inhibit the glucagon/cAMP pathway. Previous studies have demonstrated that gut microbiota-derived metabolites, including SCFAs and secondary bile acids, can directly regulate hepatic gluconeogenic gene expression through activation of G protein-coupled receptors and modulation of the gut–liver axis [25,26]. The significant increase in fecal SCFA levels in the 1 × 10^10^ CFU/day group raises the possibility that SCFA-mediated signaling may contribute to the observed suppression of hepatic gluconeogenesis, a hypothesis that warrants direct investigation in future studies. Importantly, the reduction in hepatic gluconeogenic gene expression occurred in parallel with improvements in fasting blood glucose and HOMA-IR, the latter being driven primarily by reduced fasting glucose rather than by a significant change in serum insulin. This suggests that suppression of hepatic glucose production may represent one mechanism linking the observed improvements in glycemic control to reduced hepatic glucose output.

The attenuation of hepatic TNF-α and TGF-β1 expression in DM083/163-treated mice provides additional mechanistic insight into the glycemic improvements observed. Chronic low-grade inflammation is a hallmark of obesity-associated insulin resistance, and TNF-α is a well-characterized mediator that impairs insulin receptor signaling by promoting serine phosphorylation of insulin receptor substrate-1 (IRS-1), thereby inhibiting downstream PI3K/Akt pathway activation [6]. The significant reduction in hepatic TNF-α mRNA expression, corroborated by decreased serum TNF-α concentrations, suggests that DM083/163 attenuates both local hepatic and systemic inflammation. TGF-β1 plays a dual role in metabolic disease, contributing to the progression of hepatic fibrosis and impairing insulin signaling through activation of Smad-dependent pathways [27]. The significant reduction in TGF-β1 expression in the high-dose group suggests that DM083/163 may also attenuate HFD-induced hepatic fibrogenic activity, although future studies incorporating liver histology and protein-level analysis would be necessary to confirm this interpretation. Taken together, the reductions in hepatic inflammatory markers and circulating TNF-α suggest that attenuation of chronic low-grade inflammation may have contributed to the improvement in insulin sensitivity observed in the DM083/DM163-treated mice.

Although overall beta diversity differed significantly across groups (PERMANOVA R^2^ = 0.262, *p* = 0.001), pairwise analysis indicated that this was driven primarily by the dietary contrast, and the high-dose probiotic group did not differ significantly from the HFD control (R^2^ = 0.081, *p* = 0.102). The gut microbiota changes associated with DM083/163 were therefore modest and did not constitute a statistically significant remodeling of overall community structure relative to the HFD control. Nevertheless, more targeted compositional changes, together with the increase in fecal SCFAs, may still contribute to the observed metabolic effects. The reduction in the *Bacillota*/*Bacteroidota* ratio in the high-dose group is particularly noteworthy, as an elevated *Bacillota*/*Bacteroidota* (F/B) ratio is consistently associated with obesity and metabolic dysfunction in both human and animal studies [28,29]. HFD-induced dysbiosis is characterized by a relative expansion of *Bacillota*, which are more efficient at extracting energy from dietary substrates, thereby promoting caloric overconsumption and adiposity [30]. The numerically lower F/B ratio in the DM083/163-H group, which did not reach statistical significance, may suggest a tendency toward a more favorable microbial balance; however, this non-significant trend should not be over-interpreted. Although the limited taxonomic resolution of the V3–V4 region precluded definitive genus-level identification of the administered probiotic strains in fecal samples, the observed compositional shifts and the numerically reduced F/B ratio are consistent with a modest, non-significant trend rather than a statistically robust remodeling of the gut microbial ecosystem. To further evaluate the impact of supplementation on the administered strains, endpoint fecal qPCR was performed using primers designed from whole-genome sequence information of DM083 and DM163. Probiotic-treated groups exhibited markedly lower Ct values than non-administered groups (ND, HFD, and MET) in both DM083- and DM163-targeted assays, indicating substantial enrichment of target-associated signals following supplementation (Figure 7). These findings support gastrointestinal exposure to the administered strains during the supplementation period. Nevertheless, qPCR detection alone cannot distinguish between transient gastrointestinal passage and stable gut colonization. Therefore, definitive confirmation of long-term engraftment and persistence would require longitudinal monitoring and/or shotgun metagenomic sequencing.

DM083/163 supplementation significantly increased fecal concentrations of propionic acid, butyric acid, and total SCFA levels in the 1 × 10^10^ CFU/day group compared to the HFD control group, while valeric acid showed a consistent dose-dependent increase across all probiotic groups. SCFAs, particularly butyrate and propionate, serve as critical regulators of host metabolic homeostasis through multiple mechanisms, including activation of intestinal gluconeogenesis via free fatty acid receptor 3 (FFAR3), stimulation of glucagon-like peptide-1 (GLP-1) secretion from enteroendocrine L-cells, and direct inhibition of hepatic lipogenesis [30,31]. Notably, fecal SCFA concentrations did not differ significantly between the ND and HFD control groups, suggesting that HFD feeding alone did not substantially alter SCFA production in the present model. Although HFD feeding is often associated with reduced SCFA production, previous studies have reported variable outcomes depending on diet composition, intervention duration, and microbiota adaptation. Therefore, the significant increases observed in probiotic-treated groups may reflect enhanced microbial fermentative activity induced by DM083/163 rather than restoration of HFD-induced SCFA depletion. This interpretation should be tempered by the absence of an internal standard in the SCFA quantification; although calibration performance and reproducibility supported analytical reliability, the absolute SCFA concentrations should be regarded with appropriate caution. These findings suggest that increased SCFA production may contribute to the improvement in insulin sensitivity observed in this study. Taken together, the observed changes in gut microbiota composition, increased SCFA production, reduced inflammatory signaling, and suppression of hepatic gluconeogenic gene expression are consistent with the possibility that these outcomes are mechanistically linked, although the present associative data cannot establish such links directly. SCFAs are known to influence host metabolism through regulation of intestinal barrier function, inflammatory responses, enteroendocrine hormone secretion, and hepatic metabolic pathways. Therefore, increased SCFA production may represent one plausible mechanistic link potentially connecting the gut microbial changes with reduced inflammation, decreased hepatic gluconeogenesis, and improved insulin sensitivity in DM083/163-treated mice, although this proposed pathway remains to be tested directly.

Several limitations of the present study should be acknowledged. First, the study was conducted exclusively in male C57BL/6 mice, and the sex-specific effects of DM083/163 on glycemic control remain unknown. Second, the mechanisms by which DM083/163 modulates hepatic gluconeogenesis and inflammation were not investigated at the protein level, and future studies incorporating Western blotting and immunohistochemistry would provide more definitive mechanistic insights. Third, the absence of histological analysis precludes definitive conclusions regarding the effects of DM083/163 on hepatic steatosis and fibrosis. Fourth, although 16S rRNA amplicon sequencing provided comprehensive information on microbial community composition, the functional capacity of the microbiome was not directly assessed. Shotgun metagenomic sequencing would enable a more comprehensive characterization of the functional potential of the gut microbiome in response to DM083/163 supplementation. In addition, although qPCR analysis demonstrated enrichment of DM083- and DM163-associated signals in fecal samples following supplementation, stable colonization and persistence of the administered strains were not directly evaluated. The present study evaluated the combination of DM083 and DM163 without including individual strain treatment groups. Therefore, it was not possible to determine whether the observed effects resulted from synergistic, additive, or strain-specific actions. Future studies directly comparing individual strains and their combination will be necessary to clarify the respective contributions of DM083 and DM163 to the observed metabolic benefits. Finally, the clinical translatability of the present findings requires confirmation in human intervention studies, as the effective dose of 1 × 10^10^ CFU/day in mice may not directly correspond to an equivalent human dose.

## 5. Conclusions

In this study, we demonstrated that oral administration of a probiotic combination of *Lactiplantibacillus plantarum* DM083 and *Lacticaseibacillus rhamnosus* DM163 at 1 × 10^10^ CFU/day for 12 weeks significantly improved glycemic control and insulin resistance in HFD-induced obese mice. The high-dose probiotic group showed significant reductions in fasting blood glucose, HbA1c, OGTT-derived AUC, and HOMA-IR, with efficacy broadly comparable to that observed in the metformin-treated group. These metabolic improvements were accompanied by suppression of hepatic gluconeogenic and inflammatory gene expression and increased fecal SCFA production, along with modest changes in gut microbiota composition.

Taken together, these findings suggest that DM083/163 may exert antidiabetic effects that are associated with attenuation of inflammatory responses, suppression of hepatic gluconeogenesis, and increased SCFA production. These associative findings are consistent with, but do not definitively establish, a gut–liver axis mechanism, which remains to be confirmed by future mechanistic studies. These findings support the potential of DM083/163 as a functional probiotic candidate for the dietary management of obesity-associated type 2 diabetes. Future studies incorporating protein-level mechanistic analyses, histological evaluation, functional microbiome analyses, and human clinical trials are warranted to further validate these findings and establish the translational relevance of DM083/163 supplementation.

## Figures and Tables

**Table 1:** Primer sequences and amplicon sizes used for RT-qPCR.

Gene	Forward Primer (5′–3′)	Reverse Primer (5′–3′)	Product Size (bp)
*Gapdh*	TCCAGAGACGGCCGCATCTCTT	CCAAATCCGTTCCACACCGACCTT	82
*Tnf*	GCCTCTTCTCATTCCTGCTTG	CTGATGAGAGGGAGGCCATT	115
*Tgfb1*	CGCCATCTATGAGAAAACC	GTAACGCCAGGAATTGT	190
*Pck1*	CTGCATAACGGTCTGGACTTC	CAGCAACTGCCCGTACTC	159
*G6pc*	CGACTCGCTATCTCCAAGTGA	GTTGAACCCAGTCTCCGACCA	174

*Gapdh encoding glyceraldehyde-3-phosphate dehydrogenase (GAPDH); Tnf encoding tumor necrosis factor-α (TNF-α); Tgfb1 encoding transforming growth factor-β1 (TGF-β1); Pck1 encoding phosphoenolpyruvate carboxykinase (PEPCK); G6pc encoding glucose-6-phosphatase (G6Pase).*

## Data Availability

The 16S rRNA amplicon sequencing data generated in this study have been deposited in the NCBI under BioProject accession number PRJNA1453943 and are available at https://www.ncbi.nlm.nih.gov/bioproject/PRJNA1453943 (accessed on 23 June 2026). The data will be publicly available upon publication. All other data supporting the reported re-sults are available from the corresponding author upon reasonable request.

## Supplementary Materials

The following supporting information can be downloaded at: https://www.mdpi.com/article/10.3390/nu18132107/s1, Figure S1: Gut microbiota composition at the genus level in high-fat diet-induced obese mice; Table S1: Validation parameters for SCFA quantification by gas chromatography, including calibration range, coefficients of determination (R^2^), limits of detection (LOD), and limits of quantification (LOQ); Table S2: Primer sequences and amplicon information used for DM083- and DM163-targeted qPCR analysis.

## Abbreviations

The following abbreviations are used in this manuscript:

HFD	High-fat diet
ND	Normal diet
T2DM	Type 2 diabetes mellitus
OGTT	Oral glucose tolerance test
FBG	Fasting blood glucose
HbA1c	Glycated hemoglobin
HOMA-IR	Homeostasis model assessment of insulin resistance
PEPCK	Phosphoenolpyruvate carboxykinase
G6Pase	Glucose-6-phosphatase
TNF-α	Tumor necrosis factor-alpha
TGF-β1	Transforming growth factor-beta 1
SCFA	Short-chain fatty acid
PCoA	Principal coordinates analysis
F/B ratio	*Bacillota*/*Bacteroidota* ratio
CFU	Colony-forming unit

## References

[1] Ong K.L., Stafford L.K., McLaughlin S.A., Boyko E.J., Vollset S.E., Smith A.E., Dalton B.E., Duprey J., Cruz J.A., Hagins H. (2023). Global, regional, and national burden of diabetes from 1990 to 2021, with projections of prevalence to 2050: A systematic analysis for the Global Burden of Disease Study 2021. Lancet.

[2] Khalilov R., Abdullayeva S. (2023). Mechanisms of insulin action and insulin resistance. Adv. Biol. Earth Sci..

[3] Zhu X., Chen Z., Shen W., Huang G., Sedivy J.M., Wang H., Ju Z. (2021). Inflammation, epigenetics, and metabolism converge to cell senescence and ageing: The regulation and intervention. Signal Transduct. Target. Ther..

[4] Oh K.-J., Han H.-S., Kim M.-J., Koo S.-H. (2013). Transcriptional regulators of hepatic gluconeogenesis. Arch. Pharmacal Res..

[5] Foretz M., Guigas B., Viollet B. (2023). Metformin: Update on mechanisms of action and repurposing potential. Nat. Rev. Endocrinol..

[6] Martínez Báez A., Ayala G., Pedroza-Saavedra A., González-Sánchez H.M., Chihu Amparan L. (2024). Phosphorylation codes in IRS-1 and IRS-2 are associated with the activation/inhibition of insulin canonical signaling pathways. Curr. Issues Mol. Biol..

[7] Fabregat I., Caballero-Díaz D. (2018). Transforming growth factor-β-induced cell plasticity in liver fibrosis and hepatocarcinogenesis. Front. Oncol..

[8] Mazhar M., Zhu Y., Qin L. (2023). The interplay of dietary fibers and intestinal microbiota affects type 2 diabetes by generating short-chain fatty acids. Foods.

[9] Bečić T., Jukić I., Prižmić P.Š., Matulić I., Đogaš H., Radić M., Radić J., Vuković J., Fabijanić D. (2026). Heart–gut axis in cardiometabolic disease: Microbiome-mediated pathways linking metabolic syndrome to cardiovascular risk. Medicina.

[10] Fang W., Xue H., Chen X., Chen K., Ling W. (2019). Supplementation with sodium butyrate modulates the composition of the gut microbiota and ameliorates high-fat diet-induced obesity in mice. J. Nutr..

[11] Deng M., Zhang S., Wu S., Jiang Q., Teng W., Luo T., Ouyang Y., Liu J., Gu B. (2024). Lactiplantibacillus plantarum N4 ameliorates lipid metabolism and gut microbiota structure in high fat diet-fed rats. Front. Microbiol..

[12] Yang Y., Sui J., Liao W., Wang S., Pan D., Sun G., Gao P., Xiang X., Xia H. (2026). Clinical evidence on the health benefits and safety of probiotic *Lacticaseibacillus rhamnosus*: A systematic review. Probiotics Antimicrob. Proteins.

[13] Li T., Zhang J., Niu W., Zhang X., Yuan Z., Wang L., Zhang Y., Wang L., Ji B., Qu L. (2026). Anti-obesity Effects of Lactiplantibacillus plantarum ZNFL-1 by Modulating Gut Microbiota and Lipid Metabolism in High-Fat Diet-Induced Mice. Probiotics Antimicrob. Proteins.

[14] Han M., Liao W., Dong Y., Bai C., Gai Z. (2023). Lacticaseibacillus rhamnosus Hao9 exerts antidiabetic effects by regulating gut microbiome, glucagon metabolism, and insulin levels in type 2 diabetic mice. Front. Nutr..

[15] Lee Y., Lee N.-K., Kim N., Choi Y.-M., Kim H., Paik H.-D., Park E. (2025). Lactiplantibacillus plantarum Ln4 Alleviates High-Fat Diet-Induced Obesity by Modulating Lipid Metabolism and Adipogenesis in C57BL/6 Mice. Nutrients.

[16] Kim J., Jeon S.-G., Kwak M.-J., Park S.-J., Hong H., Choi S.-B., Lee J.-H., Kim S.-W., Kim A.-R., Park Y.-K. (2024). Triglyceride-Catabolizing Lactiplantibacillus plantarum GBCC_F0227 Shows an Anti-Obesity Effect in a High-Fat-Diet-Induced C57BL/6 Mouse Obesity Model. Microorganisms.

[17] Jacouton E., Mondot S., Langella P., Bermúdez-Humarán L.G. (2023). Impact of oral administration of Lactiplantibacillus plantarum strain CNCM I− 4459 on obesity induced by high-fat diet in mice. Bioengineering.

[18] Matthews D.R., Hosker J.P., Rudenski A.S., Naylor B., Treacher D.F., Turner R. (1985). Homeostasis model assessment: Insulin resistance and β-cell function from fasting plasma glucose and insulin concentrations in man. Diabetologia.

[19] Callahan B.J., McMurdie P.J., Rosen M.J., Han A.W., Johnson A.J.A., Holmes S.P. (2016). DADA2: High-resolution sample inference from Illumina amplicon data. Nat. Methods.

[20] Livak K.J., Schmittgen T.D. (2001). Analysis of relative gene expression data using real-time quantitative PCR and the 2−ΔΔCT method. Methods.

[21] Nguyen-Phuong T., Seo S., Cho B.-K., Lee J.-H., Jang J., Park C.-G. (2023). Determination of progressive stages of type 2 diabetes in a 45% high-fat diet-fed C57BL/6J mouse model is achieved by utilizing both fasting blood glucose levels and a 2-hour oral glucose tolerance test. PLoS ONE.

[22] Lang P., Hasselwander S., Li H., Xia N. (2019). Effects of different diets used in diet-induced obesity models on insulin resistance and vascular dysfunction in C57BL/6 mice. Sci. Rep..

[23] Kim W.J., Ryu R., Doo E.-H., Choi Y., Kim K., Kim B.K., Kim H., Kim M., Huh C.S. (2025). Supplementation with the probiotic strains Bifidobacterium longum and Lactiplantibacillus rhamnosus alleviates glucose intolerance by restoring the IL-22 response and pancreatic beta cell dysfunction in type 2 diabetic mice. Probiotics Antimicrob. Proteins.

[24] Eizadi M., Salehi S.S., Rashidi M. (2025). Relationship between Changes in Serum Insulin with Hepatic Expression of PEPCK, G6Pase and HNF4α in Response to Resistance Training in Diabetic Rats with High-Fat Diet and STZ. Iran. J. Diabetes Obes..

[25] Lee H., An J., Kim J., Choi D., Song Y., Lee C.-K., Kong H., Kim S.B., Kim K. (2022). A novel bacterium, Butyricimonas virosa, preventing HFD-induced diabetes and metabolic disorders in mice via GLP-1 receptor. Front. Microbiol..

[26] Imdad S., So B., Jang J., Park J., Lee S.-J., Kim J.-H., Kang C. (2024). Temporal variations in the gut microbial diversity in response to high-fat diet and exercise. Sci. Rep..

[27] Wang X.-L., Yang M., Wang Y. (2024). Roles of transforming growth factor-β signaling in liver disease. World J. Hepatol..

[28] Karačić A., Renko I., Krznarić Ž., Klobučar S., Liberati Pršo A.-M. (2024). The association between the Firmicutes/Bacteroidetes ratio and body mass among European population with the highest proportion of adults with obesity: An observational follow-up study from Croatia. Biomedicines.

[29] Magne F., Gotteland M., Gauthier L., Zazueta A., Pesoa S., Navarrete P., Balamurugan R. (2020). The firmicutes/bacteroidetes ratio: A relevant marker of gut dysbiosis in obese patients?. Nutrients.

[30] Turnbaugh P.J., Ley R.E., Mahowald M.A., Magrini V., Mardis E.R., Gordon J.I. (2006). An obesity-associated gut microbiome with increased capacity for energy harvest. Nature.

[31] Anachad O., Taouil A., Taha W., Bennis F., Chegdani F. (2023). The implication of short-chain fatty acids in obesity and diabetes. Microbiol. Insights.

