# Supplementary material for: A Probiotic Combination of Lactiplantibacillus plantarum DM083 and Lacticaseibacillus rhamnosus DM163 Improves Glycemic Control and Insulin Resistance in High-Fat-Diet-Induced Obese Mice
Source: Nutrients. 2026 Jun 28;18(13):2107. doi: 10.3390/nu18132107 (PMC13362811; doi:10.3390/nu18132107)
Supplement: Supplementary file 1 [file nutrients-18-02107-s001.zip › nutrients-4319938_Supplementary figure legend.pdf]

Supplementary Materials: The following supporting information can be downloaded at: www.mdpi.com/xxx/s1, Figure S1: Gut microbiota composition at the genus level in high-fat diet-induced obese mice; Table S1: Validation parameters for SCFA quantification by gas chromatography, including calibration range, coefficients of determination (R^2), limits of detection (LOD), and limits of quantification (LOQ); Table S2: Primer sequences and amplicon information used for DM083- and DM163-targeted qPCR analysis.

Figure S1. Gut microbiota composition at the genus level in high-fat diet-induced obese mice.

Relative abundance of the top 11 genera detected across experimental groups, expressed as stacked bar plots. The "Unclassified" category represents ASVs that could not be assigned to a known genus under the SILVA 138.1 reference taxonomy with a bootstrap confidence threshold of 80 (minBoot = 80). It should be noted that the V3–V4 region of the 16S rRNA gene has limited taxonomic resolution within the family Lactobacillaceae; consequently, the administered probiotic strains *L. plantarum* DM083 and *L. rhamnosus* DM163 could not be unambiguously identified by amplicon-based sequencing. ND, normal diet; HFD, high-fat diet; MET, metformin (250 mg/kg/day); DM083/163-L, 1×10^9 CFU/day; DM083/163-M, 5×10^9 CFU/day; DM083/163-H, 1×10^{10} CFU/day, respectively.
