# Supplementary material for: A Probiotic Combination of Lactiplantibacillus plantarum DM083 and Lacticaseibacillus rhamnosus DM163 Improves Glycemic Control and Insulin Resistance in High-Fat-Diet-Induced Obese Mice
Source: Nutrients. 2026 Jun 28;18(13):2107. doi: 10.3390/nu18132107 (PMC13362811; doi:10.3390/nu18132107)
Supplement: Supplementary file 1 [file nutrients-18-02107-s001.zip › Table S1.pdf]

Table S1. Validation parameters for SCFA quantification by gas chromatography, including calibration range, coefficients of determination (R^2), limits of detection (LOD), and limits of quantification (LOQ).

**Analyte**	**Calibration range (μg/mL)**	**Representative R^2**	**LOD range (μg/mL)**	**LOQ range (μg/mL)**
Acetic acid	**2.441–156.25**	**0.9960**	**11.90–12.85**	**36.07–38.93**
Propionic acid	**2.441–156.25**	**0.9986**	**6.89–13.11**	**20.87–39.73**
Butyric acid	**2.441–156.25**	**0.9958**	**2.31–7.54**	**6.99–22.86**
Valeric acid	**2.441–156.25**	**0.9985**	**5.93–7.86**	**17.96–23.82**
