# Supplementary material for: A Probiotic Combination of Lactiplantibacillus plantarum DM083 and Lacticaseibacillus rhamnosus DM163 Improves Glycemic Control and Insulin Resistance in High-Fat-Diet-Induced Obese Mice
Source: Nutrients. 2026 Jun 28;18(13):2107. doi: 10.3390/nu18132107 (PMC13362811; doi:10.3390/nu18132107)
Supplement: Supplementary file 1 [file nutrients-18-02107-s001.zip › Table S2.pdf]

Table S2. Primer sequences and amplicon information used for DM083- and DM163-targeted qPCR analysis.

Target strain	Primer	Sequence (5′–3′)	Amplicon size (bp)
Lactiplantibacillus plantarum **DM083**	DM083-F	CATGAGGAGGAACCGCATTGAC	110 bp
	DM083-R	CCGGACAGCTTCTAAACCTTCC	
Lacticaseibacillus rhamnosus **DM163**	DM163-F	GCTAAGTAGGTCTCATAGGGTTGC	117 bp
	DM163-R	TCGTTGGCAAACCCCAGTTC	
