# Supplementary figures and images for: A Probiotic Combination of Lactiplantibacillus plantarum DM083 and Lacticaseibacillus rhamnosus DM163 Improves Glycemic Control and Insulin Resistance in High-Fat-Diet-Induced Obese Mice
Source: Nutrients. 2026 Jun 28;18(13):2107. doi: 10.3390/nu18132107 (PMC13362811; doi:10.3390/nu18132107)

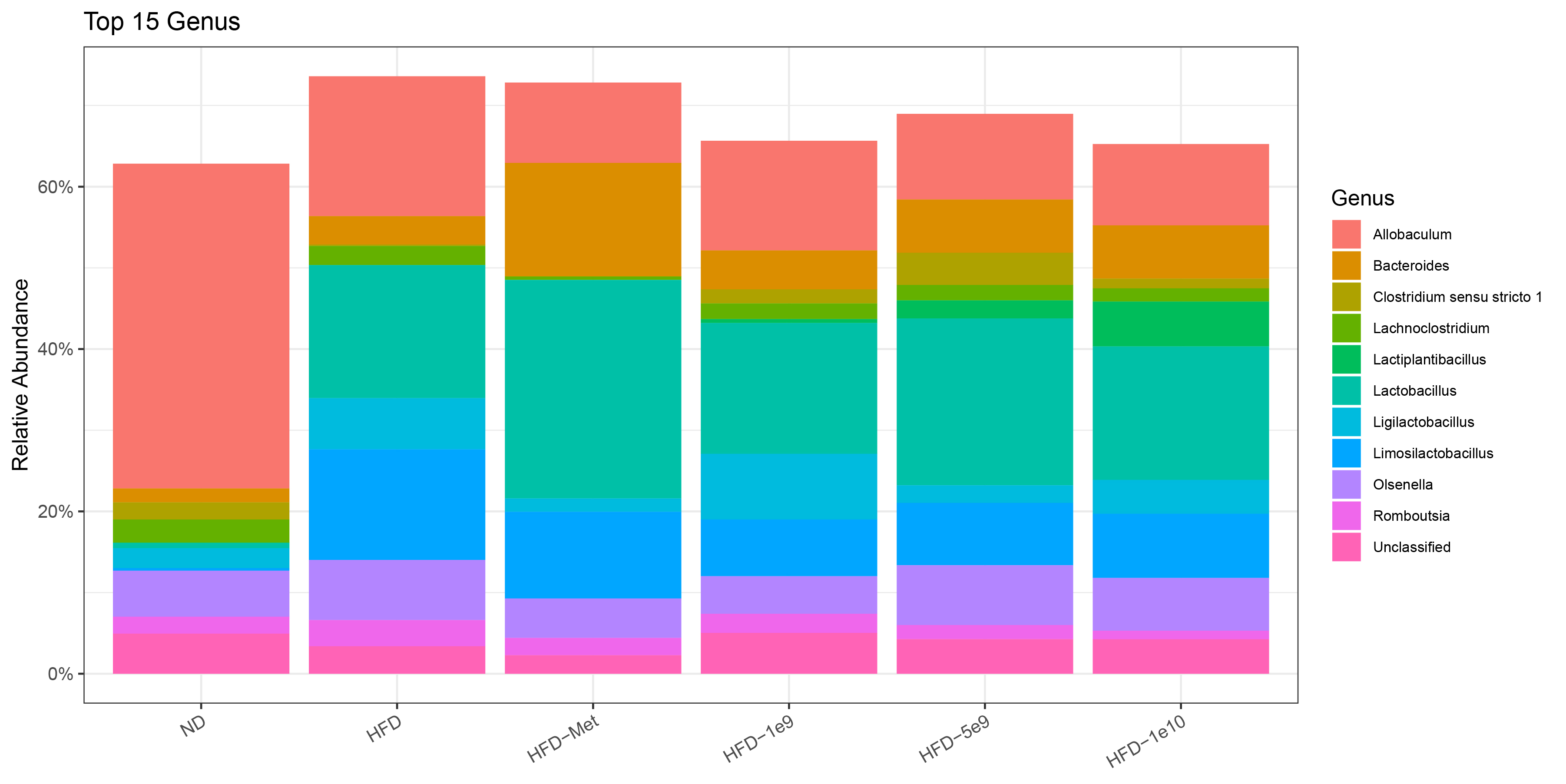

Supplement: Supplementary file 1 [file nutrients-18-02107-s001.zip › Supplementary Figure S1.jpg]
